# A Comprehensive Review on Vitamin D as a Novel Therapeutic Agent in Chronic Obstructive Pulmonary Disease

**DOI:** 10.7759/cureus.13095

**Published:** 2021-02-03

**Authors:** Amber Saleem, Shayka Sharif, Sommer Jarvis, Nikolaos Madouros, Evgenia Koumadoraki, Safeera Khan

**Affiliations:** 1 Family Medicine, California Institute of Behavioral Neurosciences & Psychology, Fairfield, USA; 2 Internal Medicine, California Institute of Behavioral Neurosciences & Psychology, Fairfield, USA; 3 Anatomy/Cell Biology, California Institute of Behavioral Neurosciences & Psychology, Fairfield, USA; 4 Surgery, California Institute of Behavioral Neurosciences & Psychology, Fairfield, USA

**Keywords:** vitamin d, copd, treatment, supplementation

## Abstract

Vitamin D has been playing an important role in the treatment of lung diseases. The non-calcemic effects of this vitamin and its role in chronic obstructive pulmonary disease (COPD) has drawn significant attention recently. Many studies have been conducted to explore the relationship between the two. We tested the hypothesis that vitamin D can act as an effective therapeutic agent for COPD by reviewing the correlation between the two and effectiveness along with the safety of supplemental vitamin D when used to treat chronic bronchitis and emphysema through clinical trials. An electronic search was conducted using combinations of keywords “vitamin D” and “COPD” from PubMed and Google scholar. Only relevant, human studies of all types were included from the last decade. A total of 36 articles were selected for review. Observational studies indicate a correlation between low serum 25(OH)D levels and obstructive lung disease pathology as well as clinical outcomes. Moreover, clinical trials were aimed to understand the impact of the use of vitamin D in improving disease indexes. These clinical trials used different drug regimes, mode of administration, and intervention duration with contrasting outcomes.

Hypovitaminosis D is a common and harmful variant of this group of obstructive lung diseases, and correcting this deficiency can improve exacerbations, inflammation, lung functions, symptoms, and quality of life. These benefits are more prevalent in patients with low baseline serum 25 hydroxyvitamin D(25(OH)D) levels. Peroral is the most frequently used route of drug administration, however, further work is required to explore the pharmacological properties of vitamin D. There was not enough literature available about the safety of the drug of intervention.

## Introduction and background

“Chronic obstructive pulmonary disease (COPD) has affected 65 million people globally and is expected to become the third most common cause of mortality by 2030 - World Health Organization” [1].

COPD is a group of obstructive airway diseases that involves chronic bronchitis and emphysema. Smoking and other air pollutants are a leading cause of the systemic inflammatory response. Host factors like genetic abnormalities, abnormal lung development, and accelerated aging also play an important role in developing the disease [2]. Tobacco smoke decreases peak lung functions and increases age-related decline [3]. Currently, inhaled corticosteroids, theophylline, and long-acting beta-2 agonist can effectively manage the disease symptoms and improve quality of life, but nothing has proven to cure the disease or reduce mortality [4]. A total of 3.2 million deaths have been reported by COPD worldwide in 2015 [5]. Mortality is directly related to acute exacerbations and systemic comorbidities of the disease [6].

Ergocalciferol (D2) and cholecalciferol (D3) are two forms of fat-soluble vitamin D. It can be obtained from diets such as fortified food, fish, and dietary supplements. Still, more than 80% of it is produced in the skin under UV radiation. In the liver, vitamin D3 is then hydroxylated to 25(OH)D, a marker for serum vitamin D levels because of its long half-life [7]. 1,25(OH)2D (calcitriol) is an active form of this vitamin formed by hydroxylation of 25(OH)D, and it is an excellent ligand for vitamin D receptor (VDR). These receptors (VDR) and vitamin D binding proteins (VDBP) are present in multiple organ systems like lungs, brain, skeletal muscle, immune cells, pancreas, breast, and colon, in addition to the organs of bone health, which plays an important role in pathophysiology behind its extraskeletal effects [8]. We are familiar with its roles in calcium, phosphorus, and bone metabolism for centuries [9]. Recently there has been increased interest in the immunomodulatory and anti-inflammatory effects of this vitamin and its correlation with chronic diseases, particularly COPD [10].

Vitamin D deficiency is a global health problem and is more common in populations with decreased sun exposure, limited mobility, and chronic diseases [11]. COPD and vitamin D share pathophysiological similarities, and many studies have shown a negative correlation between the two [12]. In relation to obstructive lung diseases and the role of supplemental calcitriol in managing disease outcomes of obstructive lung disease, hypovitaminosis D is an ongoing debate. Despite many clinical trials in this field, little is known about the efficacy, effective dosing regimes, and safety of its supplementation. The purpose of this evidence-based study is to review what impact this nutritional deficiency has on disease progression and how correcting this deficiency can help. Clinical trials from the last 10 years will be explored to find out more about the benefits and pharmacological properties of vitamin D supplements used to treat this obstructive lung disease and how baseline serum 1,25(OH)2D levels affect these trials' results. This research can help provide some evidence to the already established hypothesis that vitamin D can be a novel therapeutic agent for COPD. It will also provide grounds for new researchers to further work in required fields where more evidence is needed.

## Review

Methods

Search Method and Strategy

We conducted a thorough literature search of electronic databases PubMed, Medline, PubMed Central, Google Scholar, but PubMed remained our main database. Few articles or facts were obtained from gray literature and the web page VitaminDwiki.

A search was generated by typing combinations of keywords vitamin D, COPD, treatment, and supplementation. All articles with full literature available were included. If literature was not available for some articles, we tried to contact the author or applied institutional access to reach the subject. The number of articles by typing each keyword can be seen in Table 1.

**Table 1:** Search Results COPD: chronic obstructive pulmonary disease

Keyword	Number of articles	Database
Vitamin D	28,083	PubMed
COPD	32,759	PubMed
COPD treatment	26,725	PubMed
Vitamin D + COPD	211	PubMed

Study Selection and Data Extraction

We reviewed mixed studies (observational, experimental, review articles, and meta-analyses) to see potential effects of low serum25(OH)D levels on COPD and Clinical trials, in particular, to concentrate more on the supplemental use of vitamin D in treating COPD. Our areas of interest would be efficacy, dosing regimes, duration of therapy, and safety profile of vitamin D and how baseline serum vitamin D levels in the intervention group play with the results.

Study selection and data extraction were performed by first scanning through the titles and then by applying Inclusion exclusion criteria (Table 2) and reading through the whole articles. Some clinical trials were taken from google scholar and VitaminDwiki.

**Table 2:** Inclusion and Exclusion Criteria COPD: chronic obstructive pulmonary disease

Inclusion criteria	Exclusion criteria
Age of literature: 10 years	Studies older than 10 years
Language: English	Language other than English
Human Studies	Animal studies
Patients diagnosed with COPD	Articles with abstract only
The study should explain the correlation between serum levels of vitamin D and COPD or supplementation of vitamin D in COPD treatment	

Quality Check

The entire method was performed ethically and scientifically. Quality appraisal for individual studies and statistical analysis was not done.

Results

**Figure 1:**
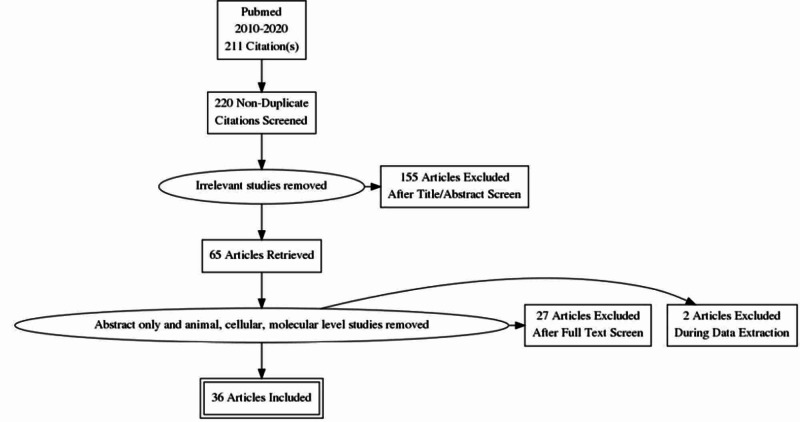
Flow Diagram for Study Selection

Search results from PubMed showed 211 articles in the last 10 years. Twenty articles were collected from other sources like google scholar, gray literature, and a web page VitaminDwiki. After removing duplicates, 220 articles were screened through the titles or abstract. One hundred and fifty-five studies were excluded, and out of 65 retrieved articles, some more studies were removed based on an animal model, cellular, or molecular levels (Figure 1).

Out of 36 final studies, 13 were observational studies performed on 48,479 disease affected patients focusing on correlation and effects of hypovitaminosis D on clinical outcomes of COPD. Three are review articles with an undefined number of participants and two meta-analyses of nine cohort studies and 25 randomized clinical trials, respectively. Regarding the benefits and role of vitamin D supplements in the treatment of COPD, we included 18 clinical trials from the last ten years that discussed mostly the effects on disease exacerbations, lung functions or spirometry indices, quality of life, muscle strength, and physical exercise. The total number of participants in these trials was 4532 (Control+ Intervention arm).

Discussion

Vitamin D and its role in extraskeletal diseases have gained enormous popularity in the last decade. Few autoimmune, respiratory, neurodegenerative, and mental health problems are among those extra skeletal problems [8]. Like its other counterparts, scientists have been trying to explore this vitamin's effects and its deficiency on obstructive lung diseases. There has been plenty of work going on in this field for years. The purpose of this study is to review the literature and figure out where we stand. Do we have enough evidence to say that this vitamin can act as a novel therapeutic agent for emphysema and chronic bronchitis?

Relationship Between Vitamin D and COPD 

**Figure 2:**
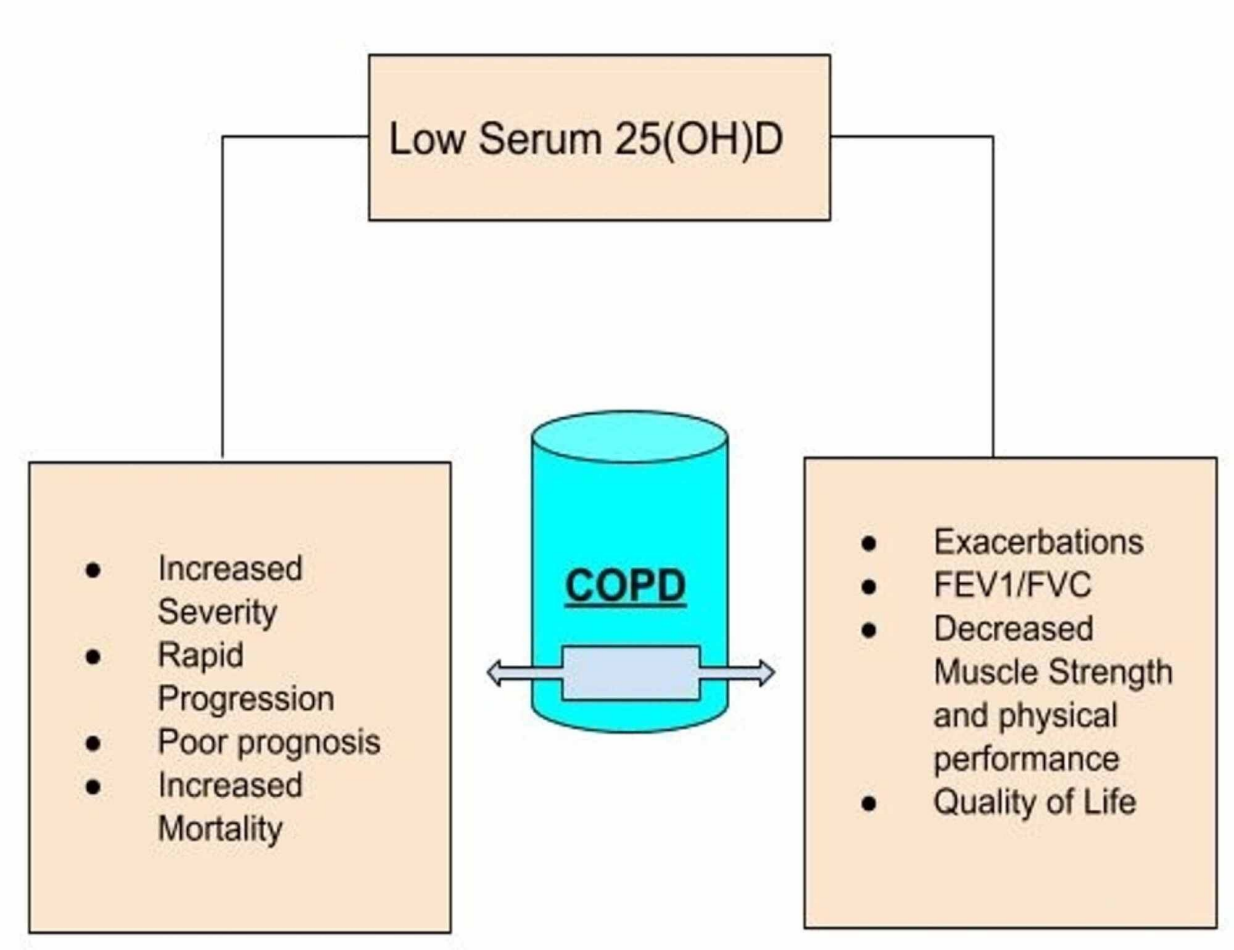
Effects of Low 25(OH)D Levels on Clinical Outcomes of COPD COPD: chronic obstructive pulmonary disease

Serum 25(OH)D levels below 20 ng/mL (50 nmol/L) is defined as vitamin D deficiency between 21-29 ng/mL (52.5-72.5 nmol/L) as insufficiency and 30 ng/mL as normal levels by US endocrinology society [13]. The prevalence of this deficiency is higher among COPD patients than in healthy control populations [14]. This, too, was more significant among patients with recent exacerbations or hospitalizations [15]. The mechanism behind this can involve decreased sun exposure due to limited mobility, aging as this group of diseases belongs to old age, chronic use of steroids that increase the breakdown of this vitamin, chronic inflammatory processes, or smoking that has a molecular relationship with the vitamin D signaling pathway [16].In light of the recently published literature, the following are the presumed effects of decreased 25(OH)D levels on the clinical outcomes of emphysema and chronic bronchitis (Figure 2). However, the search for more evidence is still ongoing.

A negative correlation has been reported between disease severity and vitamin D levels [17,18]. Kim et al. proposed that severely low 25(OH)D levels (<10 ng/mL) in male patients with COPD can increase the severity and progression of emphysema [19]. Acute exacerbation's timings and frequency were also observed to be increased under the effect of this deficiency in some studies [20,11,4], and some showed the opposite results [10]. When studied in hospitalized or chronically ill populations, an increased rate of deaths was seen among obstructive lung disease populations with severe Vitamin D deficiency, making it a poor prognostic factor with increased mortality rate and poor disease outcome [21-24]. Xu et al. conducted the largest cross-sectional meta-analysis showing a positive correlation between lung function indices (FEV1/FVC) and 25(OH)D [25]. Quality of life, muscle strength, and exercise tolerance are other disease variables with fewer observational studies. Still, all of these variables have a negative correlation with serum vitamin D levels [8,26,27].

Clinical Trials Demonstrating the Role of Supplemental Vitamin D in the Treatment of COPD

Due to the increasing desire to explore the role of vitamin D2/D3 in treating lung diseases like asthma, COPD, and cystic fibrosis, many studies have been conducted so far with inconsistent results either due to a small sample size or some other limitations. A meta-analysis of 25 randomized controlled trials (RCTs) was recently performed to see the efficacy of using this vitamin to treat emphysema and chronic bronchitis with positive results regarding acute exacerbations, lung functions, six-minute walk distance, COPD assessment score, and sputum [28]. Data extracted from our selected studies are presented in tabular form (Table 3).

**Table 3:** Data Extracted From RCTs PO: peroral; IM: intramuscular; BID: twice a day; TID: thrice a day; FEV1: forced expiratory volume; FVC: forced vital capacity; QOL: quality of life; 6MWD: six-minute walk distance; URIs: upper respiratory infections; RCT: randomized controlled trial

Author	Year of study	Study type	Sample size (n)	Variables studied	Drug dose (IU) and MOA	Length of intervention	Maintenance or additional dose	Baseline mean serum 25(OH)D	Treatment-related adverse events
Lehouck et al. [29]	2012	RCT	182	Exacerbations of COPD	100,000 PO, monthly	One year	NA	20 ng/mL	Mild, asymptomatic hypercalcemia
Hornikx et al. [30]	2012	Subgroup analysis RCT	50	Exercise performance, health-related QOL, lung functions	100,000 PO, monthly	Three months	NA	20 ng/mL	Nil
Martineau et al. [31]	2014	RCT	240	Exacerbations and URI	120,000 PO, every two months	One year	NA	Group1 (<50 ng/mL) Group 2 (50 and above)	Decreased parathyroid hormone levels
Gharabaghi et al. [32]	2013	An experimental unblended open-label trial	25	Exercise tolerance in elderly (6MWD and FEV1)	50,000 PO, weekly	Four to eight weeks	NA	Mean: 14.05 ng/mL Group 1 (<10 ng/mL) Group 2 (10–30 ng/mL)	Transient hypercalciuria (20% study population)
Khan [33]	2017	RCT	120	Exacerbations	2000, oral daily	Six months	NA	24.08 ng/mL	Not documented
Zendedel et al. [34]	2015	RCT	88	FEV1 and exacerbations	100,000 PO, monthly	Six months	NA	Not determined	Not documented
Sluyter et al. [35]	2017	RCT	442	Lung functions	200,000 PO, loading dose once	One year and one month	100,000 IU monthly	61 nmol/L	Nil
Moosavi Javad et al. [36]	2015	Before and after clinical trial	59	Lung functions	300,000–600,000 IM, loading dose once	12 weeks	50,000 IU weekly for 12 weeks	13 ng/mL	Not documented
Sanjari et al. [37]	2016	RCT	135	FEV1 and FVC	Group I: 50,000 (vitamin D3) Group II: 0.25 µg (calcitriol) PO, daily	Seven days	NA	>50 ng/mL	Nil
Alavi Foumani et al. [38]	2019	RCT	63	Spirometry findings and quality of life	50,000 PO, weekly	Eight weeks	Once a month for four months	18.55 ng/mL	Not documented
Van de Bool et al. [39]	2017	RCT	81	Exercise performance and muscle strength	6.7 µg PO, BID–TID 125 mL per serving	Four months	Other ingredients of nutritional supplement	<50 ng/mL	Stomach ache, constipation, weight loss
Bjerk et al. [40]	2013	RCT	36	Physical performance	2000 PO, daily	Six weeks	NA	23.5 ng/mL	Nil
Rafiq et al. [41]	2017	Pilot trial	50	Respiratory muscle strength and physical performance	1200 PO, daily	Six months	400 IU daily	42.3 ng/mL	Not documented
Pourrashid et al. [42]	2018	RCT	70	Quality of life in hospitalized patients with exacerbations	300,000 IM, single dose	_	NA	10–11 ng/mL	Not documented
Dastan et al. [43]	2019	RCT	70	Serum levels of systemic inflammatory biomarkers	300,000 IM, single dose	_	NA	11.25 ng/mL	Not documented
Ghodrati et al. [44]	2019	RCT	40	Dyspnea	50,000 PO, weekly	Three months	Calcium vitamin D tablet daily	<30 ng/mL	Not documented
Anjum et al. [45]	2020	RCT	30	Plasma antioxidant enzymes	80,000 PO, weekly	13 weeks 26 weeks in total	40,000IU (per one week, per two weeks, per sic weeks or no further supplementation) next 13 weeks	19.44	Not documented
Rafiq et al. [46]	2015	RCT	240	Exacerbations	16,800 PO, weekly	One year	NA	< 50 ng/mL	Study in progress

Some of the common primary outcomes of those clinical trials are exacerbations, lung functions (FEV1, FEV1/FVC), quality of life, physical exercise, and muscle strength. Lehouck’s study could not find any significant effects on the first onset of exacerbation or its frequency, quality of life, hospitalizations, and mortality after giving high dose calcitriol [29]. In comparison, Hornix et al. performed a subgroup analysis on people with very low baseline 25(OH)D levels from Lehouck's study population, which showed positive results in improving inspiratory muscle strength [30]. Later on, many RCTs proved this vitamin to be protective of the frequency of acute exacerbations of COPD [31,33,34].

Lung functions like FEV1 and FVC showed significant improvement by receiving 100,000IU of vitamin D monthly for six months, but baseline 25(OH)D levels were not recorded in this study [34]. Gharabaghi et al. and Sluyter et al. reported improved FEV1 in subgroups like vitamin D insufficient population(10-30 ng/mL) and ever smokers, particularly with lung diseases (asthma, COPD) or vitamin D deficiency respectively [32,35]. In contrast, many studies showed no significant benefits of giving supplemental vitamin D to improve lung functions [36-38]. Older people with obstructive lung diseases and decreased 25(OH)D levels often need rehabilitation exercise to improve their muscle strength and physical activity. This effect can be enhanced even more if their vitamin D levels are corrected by giving high dose supplements [29,32,39]. However, short term calcitriol supplementation and a pilot trial failed to obtain similar results [40,41].

 Both single high dose intramuscular and oral supplemental vitamin D seems to have beneficial effects on health-related quality of life [42,38]. We could not find many trials on mortality or disease prognosis as a primary outcome. Still, most of them reported no significant change seen when studied as a secondary outcome of the trials [29,42,43].

As we reviewed earlier observational studies demonstrating the role of hypovitaminosis D in disease severity, progression, poor outcome, increased inflammation, and symptoms of emphysema or chronic bronchitis, little is known from experimental studies in this field. More clinical trials are required to explore these parameters of disease in detail. Dastan et al. documented the effect of vitamin D supplements on inflammation resolution by measuring serum levels of inflammatory markers [43]. Ghodrati et al. performed a clinical trial and found out the curative role of this vitamin in treating dyspnea in COPD patients [44]. Supplementation can seemingly reduce the progression and morbidity of chronic diseases by decreasing the number of free radicals with antioxidant enzymes, as observed in the latest randomized control trial performed in Bangladesh by Anjum et al. [45]. PRECOVID and Lung VITAL are two large ongoing clinical trials with registration numbers NCT02122627 and NCT01728571, respectively. Their results might add to the evidence regarding vitamin D's effectiveness in controlling disease exacerbations and lung functions [46,47].

Pharmacologic Perspective of Supplemental Vitamin D

In RCTs, mostly the oral route of drug administration was used; only two experimented with high dose intramuscular bolus vitamin D with significant improvement in the quality of life, symptoms of acute exacerbation of COPD, and inflammation resolution [42,43]. These studies did not document any side effects related to parenteral or bolus drug regime. 

An animal trial performed on mice has put forward a new hypothesis that vitamin D, when used topically/intrapulmonary, can exert a healing effect on lung alveolar cells. This can provide a future subject to explore that inhalational can be an effective mode of administration with less systemic side effects. The healing or curative effect of this vitamin needs further human trials [48]. Transient asymptomatic hypercalcemia or hypercalciuria was reported by two studies that used high dose oral vitamin D supplements (100,000IU PO monthly and 50,000IU PO weekly) [29,32]. We couldn't find much data regarding the safety of drug intervention from our studies.

Low dose intervention performed for a shorter duration of time often failed to demonstrate beneficial effects of vitamin D on patients with emphysema or chronic bronchitis [40,37]. But these results are inconclusive because sufficient baseline 25(OH)D levels might have played along with the results [37]. In another study, vitamin D showed no potential effects on the disease parameters used in higher doses for a shorter time [36]. A huge knowledge gap exists that needs further work regarding the pharmacologic properties of calcitriol supplementation.

Effect of baseline 25(OH)D levels on results of the clinical trials: A subgroup analysis performed on 30 severely vitamin D deficient patients (serum 25(OH)D levels <10 ng/mL) raised a concern about the relationship between low baseline 25(OH)D levels and its beneficial effects on calcitriol supplementation [29]. In contrast, Gharabaghi et al. observed a significant increase in 6MWD and FEV1 in the vitamin D insufficient group (serum 25(OH)D=10-30 ng/mL) as compared to the vitamin D deficient group (serum 25(OH)D< 10 ng/mL) [32]. But later on, many studies showed similar results as Lehouck et al. and added to the hypothesis that people with low serum 25(OH)D levels benefit more from supplemental vitamin D [31,42,43]. Many authors attributed their failure to obtain the desired outcome to either inclusion of vitamin D sufficient samples or the exclusion of vitamin D deficient COPD patients [37,38,41]. Other than vitamin D deficient or insufficient population, ever smokers with hypovitaminosis D or asthma/COPD are the groups who can get more benefits from the use of vitamin D as a therapeutic agent [35]. More studies are required on larger populations involving smokers to explore this effect.

Limitations

There were only a limited number of clinical trials available that satisfied the full selection criteria. The outcomes discussed in the studies were usually widespread and only few were in the desired spectrum of interest. Side effects related to supplemental use of vitamin D were reported by only handful of studies.

## Conclusions

We reviewed the relationship between vitamin D and COPD. The efficacy, safety, and pharmacological properties of calcitriol supplements used in treating obstructive lung diseases remained our focus areas. The reviewed studies show that hypovitaminosis D is more prevalent in COPD patients, and it affects the disease symptoms, severity, progression, exacerbations, prognosis, mortality, lung functions, inflammation, and quality of life. This supplementation can reduce the number of disease exacerbations, inflammation resolution, improving physical exercise, muscle strength, and lung functions (FEV1, FEV1/FVC). In general COPD patients with vitamin D deficiency or insufficiency and smokers, benefit more from calcitriol supplementation.

Oral supplements were used most commonly, but the parenteral route also gave successful results. Some of our studies reflect that using higher doses of vitamin D for a longer duration of time gave relatively more significant results than lower doses. Still, overall results were inconclusive, and very few studies commented on the safety profile of these supplements. In the future, pharmacologic aspects and the safety of calcitriol supplements should be explored in detail. More studies are needed to talk about the alveolar healing and repairing properties of vitamin D as seen in animal models. Inhalational formulations might be the best way to administer it with the least systemic side effects however human trials are required to provide evidence. Conclusively, with the correct amount of evidence vitamin D could act as a novel curative agent for treating COPD in the future.
